# Synergistic Effects of N-Acetylcysteine and Mesenchymal Stem Cell in a Lipopolysaccharide-Induced Interstitial Cystitis Rat Model

**DOI:** 10.3390/cells9010086

**Published:** 2019-12-29

**Authors:** Jung Hyun Shin, Chae-Min Ryu, Hyein Ju, Hwan Yeul Yu, Sujin Song, Dong-Myung Shin, Myung-Soo Choo

**Affiliations:** 1 Department of Urology, Asan Medical Center, University of Ulsan College of Medicine, Seoul 05505, Korea; edwinelric17@gmail.com; 2 Department of Biomedical Sciences, Asan Medical Center, University of Ulsan College of Medicine, Seoul 05505, Korea; chaemin0427@hanmail.net (C.-M.R.); alal0903@naver.com (H.J.); hwanyel@gmail.com (H.Y.Y.); thdtnwls16@naver.com (S.S.)

**Keywords:** interstitial cystitis, mesenchymal stem cell, N-acetylcysteine, combination therapy

## Abstract

The purpose of this study was to reduce the amount of stem cells used in treating preclinical interstitial cystitis (IC model) by investigating the synergistic effects of multipotent mesenchymal stem cells (M-MSCs; human embryonic stem cell-derived) and N-acetylcysteine (NAC). Eight-week-old female Sprague-Dawley rats were divided into seven groups, i.e., sham (*n* = 10), lipopolysaccharide/protamine sulfate (LPS/PS; *n* = 10), LPS/PS + NAC (*n* = 10), LPS/PS with 25K MSC (*n* = 10), LPS/PS with 50K MSC (*n* = 10) LPS/PS + 25K MSC + NAC (*n* = 10), and LPS/PS + 50K MSC + NAC (*n* = 10). To induce the IC rat model, protamine sulfate (10 mg, 45 min) and LPS (750 μg, 30 min) were instilled once a week for five consecutive weeks via a transurethral PE-50 catheter. Phosphate-buffered saline (PBS) was used in the sham group. One week after the final instillation, M-MSCs with two suboptimal dosages (i.e., 2.5 or 5.0 × 10^4^ cells) were directly transplanted into the outer-layer of the bladder. Simultaneously, 200 mg/kg of NAC or PBS was intraperitoneally injected daily for five days. The therapeutic outcome was evaluated one week after M-MSC or PBS injection by awake cystometry and histological analysis. Functionally, LPS/PS insult led to irregular micturition, decreased intercontraction intervals, and decreased micturition volume. Both monotherapy and combination therapy significantly increased contraction intervals, increased urination volume, and reduced the residual volume, thereby improving the urination parameters compared to those of the LPS group. In particular, a combination of NAC dramatically reduced the amount of M-MSCs used for significant restoration in histological damage, including inflammation and apoptosis. Both M-MSCs and NAC-based therapy had a beneficial effect on improving voiding dysfunction, regenerating denudated urothelium, and relieving tissue inflammation in the LPS-induced IC/BPS rat model. The combination of M-MSC and NAC was superior to MSC or NAC monotherapy, with therapeutic efficacy that was comparable to that of previously optimized cell dosage (1000K) without compromised therapeutic efficacy.

## 1. Introduction

Stem cell therapy is considered a potential alternative treatment for chronic, degenerative disease which has limited therapeutic options with suboptimal efficacy [1,2,3]. Interstitial cystitis/bladder pain syndrome (IC/BPS), a symptomatic diagnosis characterized by chronic pelvic pain that lasts for more than six weeks and frequently accompanies lower urinary tract symptoms such as frequency and urgency in the absence of infection and other pathologies, is an ideal candidate for stem cell therapy [4,5,6,7]. Various treatment options, including behavioral therapy, oral pharmacotherapy, intravesical instillation, and surgical therapy, have been suggested for the treatment of IC/BPS, although none of them provide a definite cure for the disease.

In this regard, a treatment with mesenchymal stem cells (MSCs) is currently considered a new potential direction for treatment of IC/BPS in several preclinical settings [4,5,6,7,8]. The potential effects of MSCs in IC/BPS are related to their ability to produce a subset of biologically active substances with anti-inflammatory, anti-fibrosis, and tissue regenerating properties. However, the majority of the studies based on MSCs derived from adult tissues have shown critical limitations, such as the marginal and short-lasting therapeutic efficacy, which are attributed primarily to poor cell survival and engraftment at transplantation sites [9,10]. In addition, to obtain sufficient cell numbers with sustained therapeutic potency, large-scale ex vivo expansion is essential, however, it critically causes the unwanted loss of core function of stem cells and increases the cost for its production.

To overcome these issues, embryonic stem cells (ESCs) or induced pluripotent stem cells (iPSCs) have recently been employed as a promising alternative supply in order to provide sufficient cell numbers with enhanced in vivo survival, engraftment, and functionality for sustained therapeutic potency [11,12]. We recently proved the superior therapeutic effects and engraftment as well as long-term survival of human ESC-derived multipotent MSCs (M-MSCs) compared to their adult tissue counterpart, having employed several IC/BPS animal models that recapitulate the different pathogenesis by intravesical instillation of hydrochloride acid (HCl) or lipopolysaccharide/protamine sulfate (LPS/PS) as well as intravenous injection of ketamine [5,6,7].

When evaluating the usefulness of certain therapies, including stem cell therapy, safety should always be of concern along with efficacy. In particular, all the stem cell therapies with functional improvement, for example, employing ESCs or iPSCs, as well as genetic modification or chemical priming, should carefully examine the possible risk of uncontrolled differentiation or tumorigenesis after transplantation [13,14]. Both the therapeutic effect and the risk of carcinogenesis in stem cell therapy are proportional to the amount of stem cells used. In addition, the time and cost required for the isolation and expansion of stem cells increases with higher target dosage. Consequently, determining the critical concentration that provides the maximal therapeutic effect without adverse outcomes or tumorigenicity with reasonable cost is of major interest. Reducing the target dosage of stem cells via the synergistic effects of combination therapy could also be an alternative option. We recently reported the therapeutic effects of N-acetylcysteine (NAC), an anti-fibrotic agent, in the IC/BPS preclinical studies [8,15]. Therefore, this study intended to investigate whether there is a synergistic effect of M-MSCs and NAC in the LPS-induced cystitis model and to seek the possibility of reducing the amount of stem cells used in treating IC/BPS.

## 2. Materials and Methods

### 2.1. Animal Modelling

The animal experiment was approved by the Institutional Animal Care and Use Committee (IACUC-2016-12-088). Eight-week-old female Sprague-Dawley rats (OrientBio, Gapyong, Gyeonggi-do, Korea) were given intravesical PS (10 mg/rat; Sigma-Aldrich, St. Louis, MO, USA) by transurethral insertion of a 26-gauge angiocatheter in order to denude the urothelium. Forty-five minutes later, the bladder was emptied, washed with phosphate-buffered saline (PBS) solution, and LPS (750 μg/rat, Sigma-Aldrich) was instilled and maintained for 30 min so as to induce inflammation. For chronic urothelial injury, the protocol was repeated once a week for five consecutive weeks. In the sham group, PBS solution was used rather than PS and LPS.

### 2.2. Stem Cell Preparation

H9 human ESCs were differentiated into M-MSCs, as previously described [12]. Established M-MSCs were cultured in EGM2-MV medium (Lonza, San Diego, CA, USA) on the rat tail collagen type I (Sigma-Aldrich) coated plates under humidified atmosphere containing 5% CO_2_ at 37 °C. Surface marker expression and multi-lineage differentiation of M-MSCs were also characterized as previously reported [5]. All of the M-MSCs were expanded less than ten passages in order to ensure the multipotency.

### 2.3. Administration of NAC and/or M-MSCs

**Figure 1:**
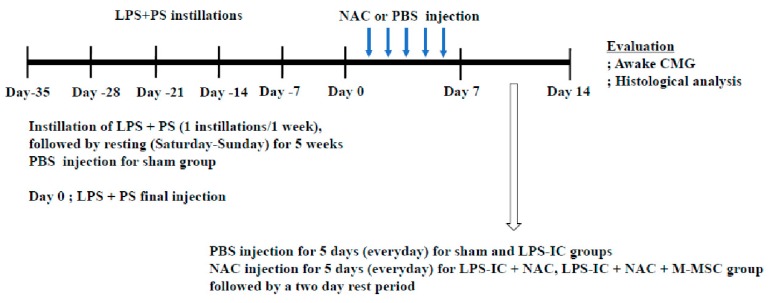
Schematic diagram of overall experiment. Ten female Sprague-Dawley rats were used in each group. The experimental control (LPS group) had PS and LPS instilled weekly for 5 weeks to induce chronic urothelial injury. Interventions involved a single administration of M-MSCs at a dose of 25K or 50K cells per 200 μL PBS into the submucosal layer of the bladder or daily intraperitoneal injection of 200 mg/kg NAC at the indicated schedules. The sham group received PBS vehicle instead of MSC or NAC injection. LPS/PS: lipopolysaccharide/protamine sulfate; IC: interstitial cystitis; M-MSCs: multipotent mesenchymal stem cells; PBS: phosphate-buffered saline; NAC: N-acetylcysteine; CMG: cystometry.

Eight-week-old, female Sprague-Dawley rats were divided into seven groups, i.e., the sham (*n* = 10), LPS (*n* = 10), LPS + NAC (*n* = 10), LPS + 25K M-MSC (*n* = 10), LPS + 50K M-MSC (*n* = 10), LPS + NAC + 25K M-MSC (*n* = 10), and LPS + NAC + 50K M-MSC (*n* = 10). One week after the final instillation of LPS/PS, a lower abdominal incision was made to expose the bladder, and the 2.5 (25K) or 5.0 (50K) × 10^4^ M-MSCs or PBS were directly injected into the outer layer of the bladder (anterior wall) using a 26-gauge needle-connected 300-μL syringe. After administration of M-MSCs, 500 μL of PBS with or without 200 mg/kg of NAC (Sigma-Aldrich) was administered intraperitoneally for five days followed by a two-day rest period (Figure 1).

### 2.4. Unanesthetized and Unrestrained Cystometry

Bladder function was evaluated two weeks after the final instillation of LPS/PS under unrestrained and awake state in metabolic cages, as previously described [5,6,7,15].

The parameters used for awake cystometric analysis were as follows: an increase in intravesical pressure above 15 cm H_2_O from the baseline without recorded voiding volume was defined as non-voiding contraction (NVC). Basal pressure (BP) was the lowest bladder pressure value during the filling phase, and micturition pressure (MP) was the maximum bladder pressure during the voiding phase. Bladder capacity (BC) was the total amount of saline infusion, micturition volume (MV) was the amount of voided urine recorded by the fluid collector, and the residual volume (RV) was calculated as BC-MV. The micturition interval (MI) was the interval between each micturition. The mean values from three reproducible voiding cycles in individual animals were used for analysis.

### 2.5. Histological Examinations

Immediately after the awake cystometry, the bladder tissue was harvested for histological analysis, which evaluated the epithelial denudation, mast cell infiltration, tissue fibrosis, and apoptosis with cytokeratin immunostaining (Keratin, Pan Ab-1; Thermo Scientific, Foster City, CA, USA), toluidine blue staining (Toluidine blue-O; Daejung Chemicals and Metals, Seoul, Korea), Masson’s trichrome staining (Junsei Chemical, Tokyo, Japan), and terminal deoxynucleotidyl transferase dUTP nick end labeling (TUNEL) staining (Roche, Mannheim, Germany), respectively [6,7,16]. Engraftment of the administered M-MSCs was determined by immunofluorescence analysis (EVOS, FL Color Imaging System, Life Technologies, Carlsbad, CA, USA) of human β2-microglobulin (hB2M) (mouse monoclonal, SC80668; Santa Cruz Biotechnology Inc., Paso Robles, CA, USA) and visualized by Alexa 488-conjugated (A11029) anti-mouse antibody (Molecular Probes, Grand Island, NY, USA), as previously described [2,9]. The nuclei were counterstained with 4′,6′-diamino-2-phenylindole. Quantitative digital image analysis was performed in three randomly selected representative areas of each slide from five independent animals using Image-Pro 5.0 software (Media Cybernetics, Rockville, MD, USA).

### 2.6. Statistical Analysis

Data were reported as the mean ± standard error of the mean (SEM) and were analyzed using GraphPad Prism 7.0 (GraphPad Software, La Jolla, CA, USA). Differences and significance were verified using 1-way or 2-way ANOVA followed by Bonferroni post-hoc tests. In this study, *p*-values of <0.05 were considered statistically significant.

## 3. Results

### 3.1. Therapeutic Efficacy of NAC or M-MSC Monotherapy

We have previously identified several IC/BPS animal models that could summarize the different pathogenesis by intravesical instillation of hydrochloride acid (HCl) for an acute model or instillation of PS and LPS for a chronic model, as well as the short- or long-term intravenous injection of ketamine for ketamine-induced cystitis (KC). In these animal models of IC/BPS or KC, the therapeutic efficacy of hESC-derived M-MSCs was superior to that of adult bone marrow-derived stem cells, and did not have any adverse outcomes such as abnormal growth, tumorigenesis, or immune-mediated transplant rejection during the 12 months post-injection [5,6,7]. However, a considerable amount of M-MSCs of approximately 1.0 × 10^6^ M-MSCs should be optimized in order to improve the safety and cost-effectiveness of the advanced MSC therapy based on human ESCs. To address this issue, we first examined whether a single local administration of M-MSCs with a reduced cell number (50 × 10^3^; 50K) into the bladder could be effective for ameliorating the bladder voiding function in the chronic IC/BPS animal model in which instillation of PS and LPS into the bladders of rats at once per week for five weeks induces longer lasting and chronic urothelial injury [6,17]. We also examined the therapeutic effect of a daily intraperitoneal injection of NAC, which was beneficial to LPS-induced cystitis or to KC in our previous preclinical studies [8,15] due to its antifibrotic activity.

**Figure 2:**
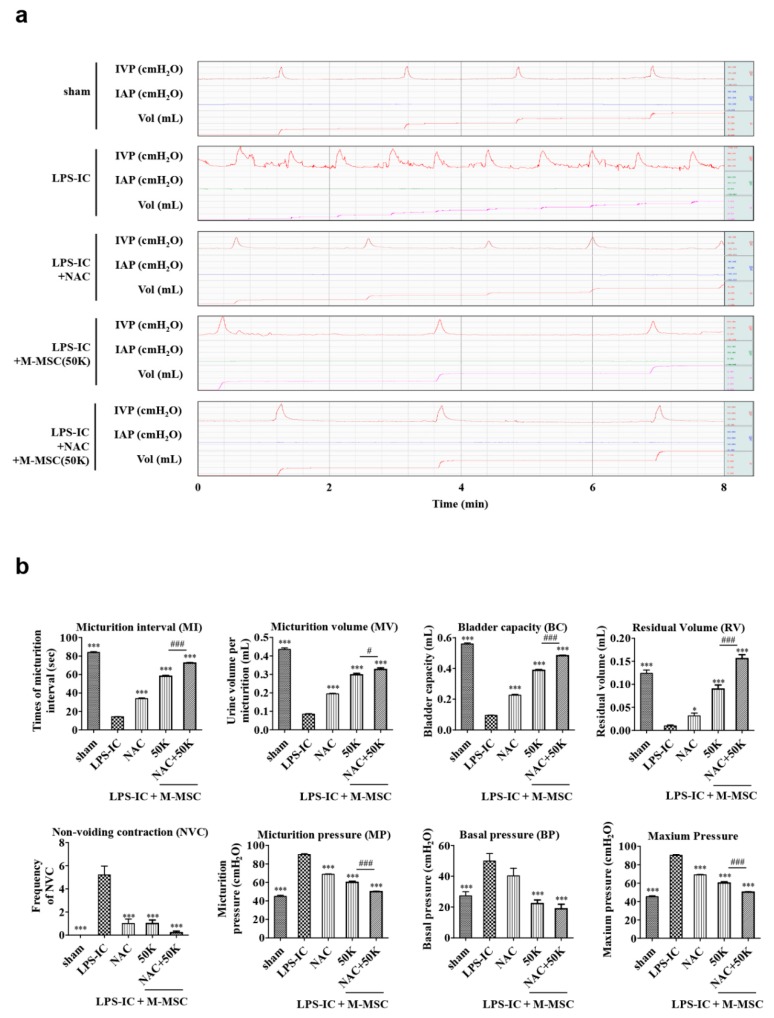
N-acetylcysteine (NAC) and 50K M-MSCs injection ameliorated the bladder voiding function in a LPS-IC rat. (**a**) Awake cystometry. (**b**) Representative awake cystometry results and quantitative bladder voiding parameters at one week after NAC and M-MSCs (50K, K = thousand) injection. The data are presented as the mean ± standard error of the mean (from five independent animals in each group). *p < 0.05, **p < 0.01, ***p < 0.001 compared with the LPS group using the Bonferroni post-test. IVP: intravesical pressure; IAP: intra-abdominal pressure; sham, sham operated.

In line with a previous report [6], animals with LPS and PS instillation showed increased non-voiding contraction, basal and micturition pressure, as well as decreased micturition interval, bladder capacity, micturition volume, and residual volume (Figure 2a,b, Figure S1). Monotherapy with a single administration of 50K M-MSCs or a daily injection of NAC ameliorated the defects in bladder voiding functions that were observed in the LPS-induced IC/BPS rat model (Figure 2a,b, Figure S1). More importantly, a single administration of 50K M-MSC had the most superior therapeutic effects rather than those of the daily injection of NAC monotherapy.

### 3.2. Improved Therapeutic Efficacy of NAC and M-MSC Combination Therapy

Both monotherapy with 50K M-MSC or NAC showed unsatisfactory therapeutic outcomes compared with the previous report in which 1 × 10^6^ M-MSCs were locally administrated [6]. In particular, the frequency of contraction during the non-voiding period (non-voiding contraction; NVC), the most common factor for evaluating bladder voiding function in clinical care, was still observed in the LPS-IC rats who were given the 50K or NAC monotherapy (Figure 2b). Therefore, we examined whether the combination of 50K M-MSC and NAC treatments could synergistically improve the therapeutic outcomes in the LPS-IC rat model. As shown in Figure 2a,b, the combination therapy of 50K M-MSC and NAC had a superior therapeutic efficacy than the corresponding monotherapies. In particular, the 50K M-MSC and NAC combination therapy significantly ameliorated the frequency of NVC, as was similar to the optimal M-MSC therapy with 1 × 10^6^ cells.

**Figure 3:**
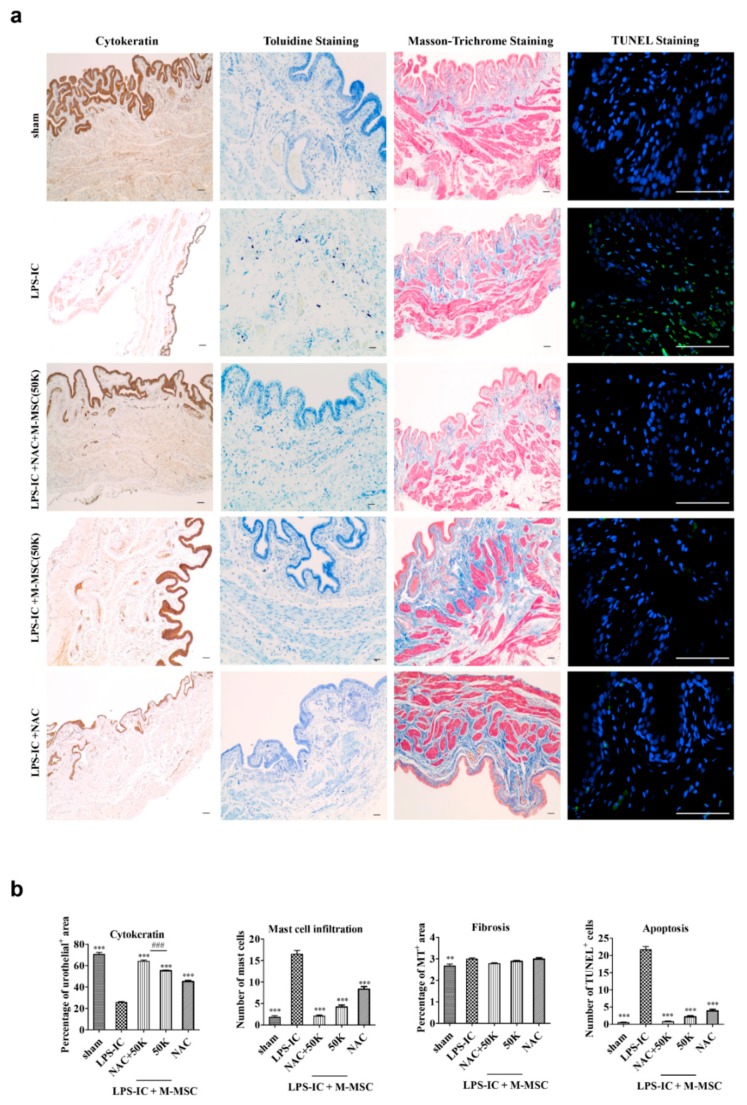
Injection of NAC and 50K M-MSCs ameliorated histological abnormalities in the bladders of LPS-IC rats. (**a**) (i) Cytokeratin immunostaining (magnification ×40, scale bar = 200 μm), (ii) Toluidine blue staining (magnification ×100, scale bar = 200 μm), (iii) Masson’s trichrome staining (magnification ×100, scale bar = 200 μm), and (iv) TUNEL (magnification ×400, scale bar = 100 μm) of bladder tissue of LPS-IC rats at one week after injection of the indicated number of NAC or M-MSCs (50K, K = thousand) or PBS. The arrows indicate infiltrated mast cells (ii) or apoptotic cells (iv). sham: sham-operated. (**b**) Quantification of the histological staining. Three representative areas per slide were randomly selected from five independent animals. Data (*n* = 15) were normalized against those in sham-operated rats and are presented as the mean ± SEM. **p < 0.01 and ***p < 0.001 were compared with the LPS-IC group; #*p* < 0.05, ##*p* < 0.001, and ###*p* < 0.001 were compared with the NAC + 50K group according to a one-way ANOVA using the Bonferroni post-hoc test.

We next evaluated the beneficial effects of 50K M-MSC or NAC monotherapy and combination therapy on the histological features observed in IC patients. The bladder tissues in the LPS-IC group were characterized by (i) severe loss of the cytokeratin expressing urothelium, (ii) the infiltration of Toluidine blue-stained mast cells, and (iii) the increase of TUNEL-stained apoptotic cells (Figure 3a,b). Masson’s trichrome staining revealed that tissue fibrosis was little affected in the bladders of LPS-IC rats. In line with the findings of the awake cystometric analysis, combination therapy with 50K M-MSC and NAC effectively restored denuded urothelium and decreased mast cell infiltration and apoptosis. In particular, the urothelial damages were more successfully repaired by the 50K and NAC combination therapy compared with that of its monotherapy. Tissue fibrosis in the bladder was slightly increased in the LPS-IC group, although without statistical significance, as previously reported [6]. Taken together, these results demonstrate that the single local administration of M-MSCs with a reduced number can ensure the therapeutic outcome when the recipient has been conditioned with NAC.

### 3.3. Optimization of the M-MSC and NAC Combination Therapy

**Figure 4:**
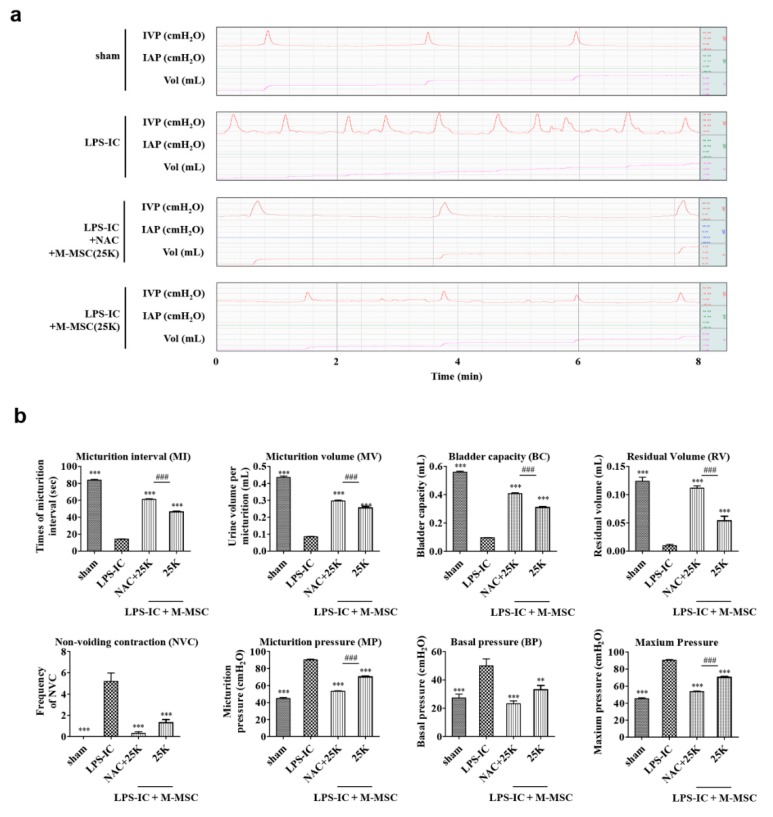
Injection of NAC and 25K M-MSCs ameliorated bladder voiding functions in LPS-IC rats. (**a**) Representative awake cystometry results (**b**) and quantitative bladder voiding data at one week after injection of LPS-IC rats with the indicated number of NAC and M-MSCs (25K, K = thousand). Data are presented as the mean ± SEM (five independent animals per group). *p < 0.05, **p < 0.01, and ***p < 0.001 were compared with the LPS-IC group and #*p* < 0.05, ##*p* < 0.001, and ###*p* < 0.001 were compared with the NAC + 25K group according to a one-way ANOVA using the Bonferroni post-hoc test. sham: sham-operated.

Next, we further reduced the number of M-MSCs for combination therapy with NAC, as much as up to 25 × 10^3^ (25K), which was one-fortieth of that of the previous preclinical report [6]. The combination therapy of 25K M-MSC and NAC had superior therapeutic efficacy to that of NAC or 25K M-MSC monotherapy (Figure 4a,b, Figure S1). Compared with monotherapy with 25K M-MSC or NAC, the combination therapy presented a significant improvement in bladder voiding functions regarding the micturition interval, micturition volume, residual volume, basal pressure, bladder capacity, and NVC (Figure 4b, Figure S1).

**Figure 5:**
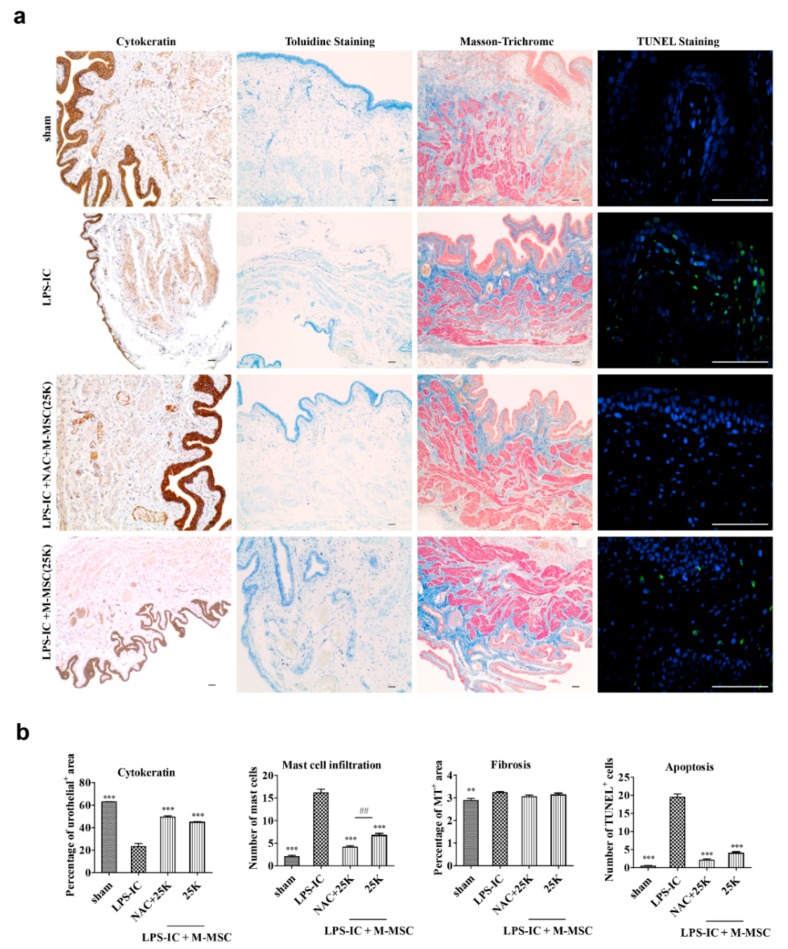
Histological analysis of the effect of N-acetylcysteine (NAC) and M-MSCs pharmacologic therapy on the bladder of the lipopolysaccharide-induced cystitis (LPS) rat model. (**a**) The histological analysis of urothelium, mast cell infiltration, fibrosis, and apoptosis in the bladder tissues of the indicated groups. Nuclei were stained with 4′,6′-diamino-2-phenylindole (blue). (**b**) Quantitative data of each staining are presented on the right side of the indicated representative pictures. All of the quantitative data were normalized to those of the sham group and are presented as a dot plot with a mean ± standard error of the mean (*n* = 15). *p < 0.05, **p < 0.01, and ***p < 0.001 compared with the LPS using the Bonferroni post-test.

Consistent with awake cystometry data, 25K M-MSC and NAC combination therapy effectively restored histological alterations in LPS-IC rats, such as severe urothelial denudation, mast cell infiltration, and an increase in apoptosis (Figure 5). More importantly, the therapeutic outcomes for the IC/BPS characteristic histological injuries were superior in the 25K M-MSC and NAC combination therapy compared with monotherapy with 25K M-MSC.

### 3.4. Distribution of Transplanted M-MSCs

**Figure 6:**
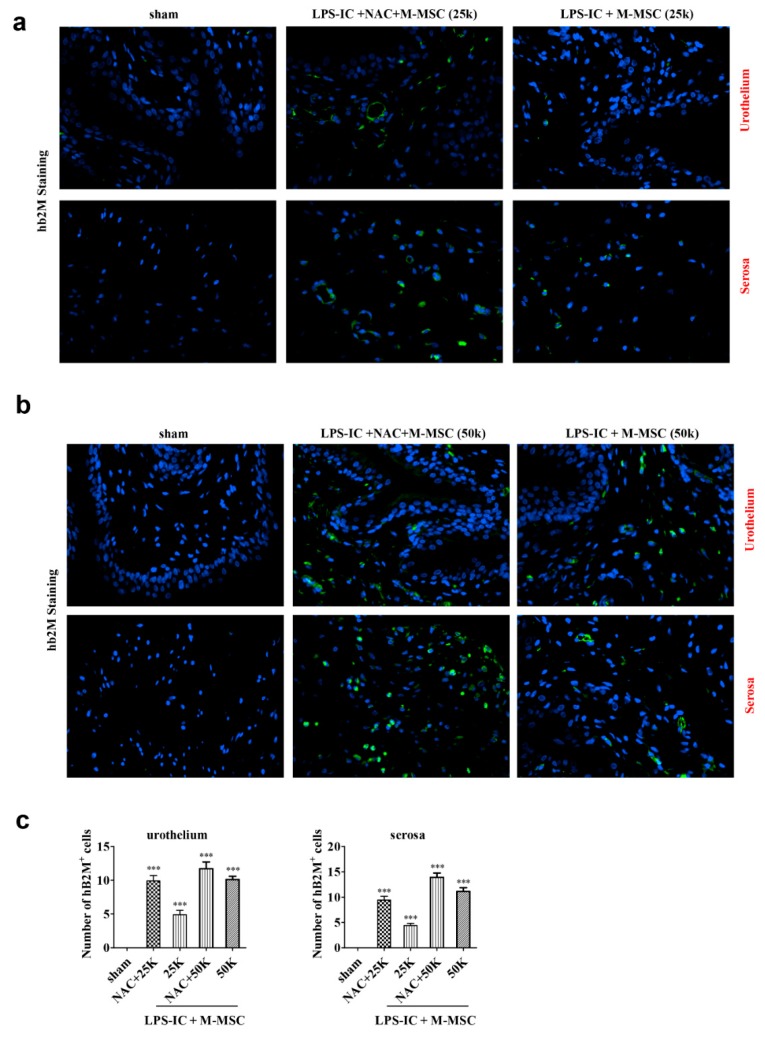
Human antigens in transplanted M-MSCs. (**a**) Representative immunofluorescence micrographs of bladder sections of LPS-IC + 25K M-MSC rats stained for hB2M (green) structures (magnification ×1000, scale bar = 10 μm). (**b**) Representative immunofluorescence micrographs of bladder sections of LPS-IC + 50K M-MSC rats stained for hB2M (green) structures (magnification ×1000, scale bar = 10 μm). Nuclei were stained with DAPI (blue). (**c**) Number of transplanted M-MSCs (hB2M positive cells) in urothelium and serosa. DAPI: 4’,6-diamidino-2-phenylindole

Transplanted M-MSCs were observed in both the urothelium and the serosa and with a slight dominance in the urothelium. The combination therapy with NAC resulted in a greater number of transplanted cells than the same dose of M-MSC monotherapy. Moreover, the combination of NAC and 25K M-MSC presented similar counts of hB2M cells to those of 50K M-MSC monotherapy (Figure 6).

## 4. Discussion

In this study, we demonstrated the synergistic effect of the NAC and M-MSC combination in a LPS-induced cystitis rat model. Concurrent NAC treatment further improved bladder function and histology more than M-MSC monotherapy. The combination of NAC and 25K M-MSC presented a similar degree of functional and histological restoration to 50K M-MSC monotherapy. In addition, combination therapy with NAC and stem cell resulted in a greater number of transplanted stem cells in both the urothelium and serosa of rat bladder.

Among studies that have reported the therapeutic efficacy of stem cells in different preclinical models, only one study focused on the synergism of melatonin and mesenchymal stem cells [18]. Chen et al. made a cyclophosphamide-induced cystitis rat model with adult Sprague-Dawley rats and compared the protective effects in intravenous administration of 1.2 × 10^6^ adipose-derived mesenchymal stem cells, melatonin, and both by 24 h urine analysis (total volume, albumin level, and hematuria) and bladder tissue analysis. The increased number of inflammatory biomarkers (i.e., CD 14+. CD 74+, Cox-2+, interleukin (IL)-6, IL-12, tumor necrosis factor (TNF)-a, etc.) and reactive oxygen species (i.e., NOX-1, NOX-2, NOX-4) in the cyclophosphamide rat model was diminished in the stem cell, melatonin, and combination group. Antioxidant expressions in bladder tissue (GR, GPx, HO-1, NQO 1) were prominently increased in the combination group more than in the monotherapy group. The combined regimen of melatonin and stem cells provided superior protection against cyclophosphamide-induced cystitis in the rat model.

Melatonin and its metabolites are potent anti-inflammatory agents and free radical scavengers, and melatonin has up to ten-fold potency compared with vitamin E in regard to oxidative stress. Free radicals such as reactive nitrogen species and reactive oxygen species are efficiently neutralized by melatonin. Several experiments have demonstrated protective effects of melatonin in ischemia-reperfusion injury, which commonly occurs in myocardiac infarction and partial nephrectomy. Melatonin also stabilizes cell membranes, making them less susceptible to oxidative stress [19,20]. Similarly, NAC is also a well-known antioxidant that directly scavenges oxygen-free radicals and inhibits fibrosis. In our previous study, NAC treatment blocked the activation of the TGF-β pathway in which genes such as Cxcl10 (pro-inflammatory) and Card 10 (apoptosis-related) were down-regulated [15].

There may be a concern that if NAC monotherapy is sufficient then why should there be efforts to apply stem-cell therapy despite the risk of carcinogenesis. NAC presented comparable therapeutic effects to 25K M-MSC monotherapy in an LPS-induced cystitis rat model. However, both of them only alleviated the functional and histological deterioration to certain degrees, and the extent of the alleviation was, by far, too insufficient compared to the higher dose of stem-cell therapy, combination therapy, and ultimately sham. In addition, clinical application of NAC in bladder disorders has a long way to go, as the administration route and dosage have not been established. This should, however, be done with caution, as certain concentrations of NAC have shown pro-oxidative action, and a high dose of NAC increased abnormal cell proliferation, which resulted in papillary urothelial hyperplasia in previous studies [21].

The main limitation of this study is that we only evaluated bladder function and histology to determine the therapeutic effect and did not confirm the mode of action. However, the exact pathophysiology of interstitial cystitis is also unclear and considered multifactorial.

In conclusion, we demonstrated that both MSCs and NAC-based therapy had beneficial effects in restoring voiding dysfunction, regenerating denudated urothelium, and relieving tissue inflammation in the LPS-induced IC rat model. In addition, the combination of M-MSC and NAC was superior to MSC or NAC monotherapy, with therapeutic efficacy comparable to our previous exam with 1000K MSCs. Combination therapy presented a synergistic therapeutic effect and the stem cell dosage used in the LPS model was reduced to one-fortieth.

## Supplementary Materials

The following is available online at https://www.mdpi.com/2073-4409/9/1/86/s1: Figure S1: Administration of NAC and M-MSCs improved the bladder function.
