# Supplementary material for: Synergistic Effects of N-Acetylcysteine and Mesenchymal Stem Cell in a Lipopolysaccharide-Induced Interstitial Cystitis Rat Model
Source: Cells. 2019 Dec 29;9(1):86. doi: 10.3390/cells9010086 (PMC7017055; doi:10.3390/cells9010086)
Supplement: Supplementary file 1 [file cells-09-00086-s001.pdf]

# Supplementary Material

# Synergistic Effects of N-Acetylcysteine and Mesenchymal Stem Cell in a Lipopolysaccharide-Induced Interstitial Cystitis Rat Model

**Jung Hyun Shin [1,†], Chae-Min Ryu [2,†], Hyein Ju [2,†], Hwan Yeul Yu [2], Sujin Song [2], Dong-Myung Shin [2,*] and Myung-Soo Choo [1,*]**

[1] Department of Urology, Asan Medical Center, University of Ulsan College of Medicine, Seoul 05505, Korea; edwinelric17@gmail.com

[2] Department of Biomedical Sciences, Asan Medical Center, University of Ulsan College of Medicine, Seoul 05505, Korea; chaemin0427@hanmail.net (C.-M.R.); alal0903@naver.com (H.J.); hwanyel@gmail.com (H.Y.Y.); thdtnwls16@naver.com (S.S.)

* Correspondence: d0shin03@amc.seoul.kr (D.-M.S.); mschoo@amc.seoul.kr (M.-S.C.); Tel.:+ 82-2-3010-2086 (D.-M.S.); +82-2-3010-3735 (M.-S.C.); Fax: +82-2-3010-8493 (D.-M.S.); +82-2-477-8928 (M.-S.C.)

† These authors contributed equally to this work.

## SUPPLEMENTARY FIGURE LEGENDS

| Group (n=10) | MP ($cmH_2O$) | BP ($cmH_2O$) | BC (mL) | MV (mL) | RV (mL) | MI (sec) |
|---|---|---|---|---|---|---|
| sham | 44.86 ± 7.81 | 27.32 ± 8.55 | 0.55 ± 0.04 | 0.43 ± 0.06 | 0.12 ± 0.05 | 83.80 ± 6.07 |
| LPS-IC | 90.17 ± 6.53 | 49.87 ± 15.38 | 0.09 ± 0.01 | 0.08 ± 0.02 | 0.01 ± 0.02 | 14.19 ± 2.23 |
| LPS-IC + NAC | 68.80 ± 3.53 | 40.25 ± 15.67 | 0.22 ± 0.04 | 0.19 ± 0.02 | 0.03 ± 0.04 | 33.86 ± 5.65 |
| LPS-IC + M-MSC 25K | 70.09 ± 4.96 | 33.12 ± 9.45 | 0.30 ± 0.03 | 0.26 ± 0.04 | 0.05 ± 0.41 | 46.38 ± 4.06 |
| LPS-IC + M-MSC 50K | 59.97 ± 6.49 | 22.29 ± 7.17 | 0.38 ± 0.03 | 0.30 ± 0.04 | 0.09 ± 0.05 | 58.04 ± 3.88 |
| LPS-IC + M-MSC 25K + NAC | 53.36 ± 4.08 | 23.19 ± 6.22 | 0.40 ± 0.03 | 0.30 ± 0.04 | 0.11 ± 0.03 | 61.08 ± 5.12 |
| LPS-IC + M-MSC 50K + NAC | 49.91 ± 2.60 | 18.83 ± 9.42 | 0.48 ± 0.02 | 0.33 ± 0.05 | 0.16 ± 0.05 | 72.33 ± 2.80 |

**Figure S1.** Administration of NAC and M-MSCs improved the bladder function. The micturition pressure (MP), basal bladder pressure (BP), bladder capacity (BC), micturition volume (MV), residual volume (RV), and micturition interval (MI) were quantified from the voiding pattern analysis.
